# Incidence of WHO Stage 3 and 4 Conditions following Initiation of Anti-Retroviral Therapy in Resource Limited Settings

**DOI:** 10.1371/journal.pone.0052019

**Published:** 2012-12-20

**Authors:** Andrea J. Curtis, Catherine S. Marshall, Tim Spelman, Jane Greig, Julian H. Elliot, Leslie Shanks, Philipp Du Cros, Esther C. Casas, Marcio Silveria Da Fonseca, Daniel P. O’Brien

**Affiliations:** 1 Department of Epidemiology and Preventive Medicine, Monash University, Melbourne, Australia; 2 Infectious Diseases Unit, Alfred Hospital, Melbourne, Australia; 3 Centre for Population Health, Burnet Institute, Melbourne, Australia; 4 Manson Unit, Médecins Sans Frontières, London, United Kingdom; 5 Public Health Department, Médecins Sans Frontières, Amsterdam, The Netherlands; 6 Department of Infectious Diseases, Geelong Hospital, Geelong, Australia; 7 Department of Medicine and Infectious Diseases, Royal Melbourne Hospital, University of Melbourne, Melbourne, Australia; Vanderbilt University, United States of America

## Abstract

**Objectives:**

To determine the incidence of WHO clinical stage 3 and 4 conditions during early anti-retroviral therapy (ART) in resource limited settings (RLS).

**Design/Setting:**

A descriptive analysis of routine program data collected prospectively from 25 Médecins Sans Frontières supported HIV treatment programs in eight countries between 2002 and 2010.

**Subjects/Participants:**

35,349 study participants with median follow-up on ART of 1.33 years (IQR 0.51–2.41).

**Outcome Measures:**

Incidence in 100 person-years of WHO stage 3 or 4 conditions during 5 periods after ART initiation. Diagnoses of conditions were made according to WHO criteria and relied upon clinical assessments supported by basic laboratory investigations.

**Results:**

The incidence of any WHO clinical stage 3 or 4 condition over 3 years was 40.02 per 100 person-years (31.77 for stage 3 and 8.25 for stage 4). The incidence of stage 3 and 4 conditions fell by over 97% between months 0–3 and months 25–36 (77.81 to 2.40 for stage 3 and 28.70 to 0.64 for stage 4). During months 0–3 pulmonary tuberculosis was the most common condition diagnosed in adults (incidence 22.24 per 100 person-years) and children aged 5–14 years (25.76) and oral candidiasis was the most common in children <5 years (25.79). Overall incidences were higher in Africa compared with Asia (43.98 versus 12.97 for stage 3 and 8.98 versus 7.05 for stage 4 conditions, p<0.001). Pulmonary tuberculosis, weight loss, oral and oesophageal candidiasis, chronic diarrhoea, HIV wasting syndrome and severe bacterial infections were more common in Africa. Extra-pulmonary tuberculosis, non-tuberculous mycobacterial infection, cryptococcosis, penicilliosis and toxoplasmosis were more common in Asia.

**Conclusions:**

The incidence of WHO stage 3 and 4 conditions during the early period after ART initiation in RLS is high, but greatly reduces over time. This is likely due to both the benefits of ART and deaths of the sickest patients occurring shortly after ART initiation. Access to appropriate disease prevention tools prior to ART, and early initiation of ART, are important for their prevention.

## Introduction

Efforts to scale up provision of anti-retroviral therapy (ART) in resource-limited settings (RLS) have dramatically increased over the past decade [1], [2]. A vital role of such programs is diagnosis, treatment and prevention of HIV associated conditions which contribute significantly to morbidity and mortality in patients on ART in RLS [2], [3], [4], [5].

In developed countries ART reduces the incidence of HIV associated conditions [6], [7], [8], [9]. In RLS there is a lack of data regarding the frequency and relative importance of common HIV associated conditions after commencement of ART. It is also unclear whether there are differences in disease incidence rates between regions and age groups.

A more detailed understanding of the incidence of specific HIV associated conditions after ART initiation will aid clinicians in management of patients during ART and assist program planners in strategic decision making and resource allocation for prevention, diagnosis and treatment of these conditions. Therefore, this study aims to describe the incidence of WHO Stage 3 and 4 conditions for three years following ART initiation in patients attending Médecins Sans Frontières (MSF) supported treatment programs in RLS and to examine how this is influenced by region and age.

## Methods

### Ethics Statement

The Monash University Human Research Ethics Committee, Alfred Health Human Ethics Committee, and MSF Human Ethics committee approved the study.

### Study Setting and Population

We analysed routine clinical data collected prospectively between 2002 and 2010 from 25 MSF supported HIV treatment programs in ten countries (Democratic Republic of Congo, Ethiopia, India, Ivory Coast, Moldova, Myanmar, Nigeria, Republic of Congo, Zambia, and Zimbabwe).

Adults and children who commenced ART without previously taking antiretrovirals and could contribute at least 3 months of follow-up data were eligible for inclusion. Patients were stratified into the following age groups: <5 years, 5–14 years, ≥15 years. Children <2 years of age with unknown HIV status who were receiving ART were excluded.

HIV associated clinical conditions diagnosis and treatment protocols, adherence counselling, patient follow-up, data collection and monitoring, laboratory protocols, and drug procurement and supply mechanisms were standardised across MSF programmes [10]. Treatment sites included hospital settings and peripheral health clinics.

Diagnoses of HIV-associated clinical conditions were usually made according to WHO criteria for individual conditions [11] by treating clinicians (medical doctors and clinical officers). Diagnoses relied on clinical assessments supported by basic laboratory investigations, including biochemistry, haematology, sputum microscopy for acid fast bacilli (AFB) stains, India ink examination of cerebro-spinal fluid and chest x-ray. More sophisticated investigations such as cryptococcal antigen assay and abdominal ultrasound were available at limited sites.

ART regimens were usually non-nucleoside reverse-transcriptase inhibitor (NNRTI) based. Eligibility criteria for ART and first-line regimens were standardized and based on WHO guidelines [11], [12]. Clinical consultations and intensive adherence counselling sessions were provided before and during ART. Daily co-trimoxazole prophylaxis was given to all patients with clinical stage 2, 3 and 4 disease or those with CD4 cell count <350 cells/mm^3^. Patients did not routinely receive prophylaxis for tuberculosis (TB) or Mycobacterium Avium Complex (MAC), apart from those in Myanmar programmes where MAC prophylaxis was generally provided for patients with CD4<50 cells/mm^3^. CD4 counts were usually monitored using automated methods but HIV viral load was not systematically monitored. All treatment was free.

### Data Collection

Information about medical history and sociodemographic characteristics were collected at the initial consultation. Clinical and therapeutic information, including any new HIV associated clinical conditions, weight, and duration of follow up was recorded at subsequent consultations. All information was collected on standard forms and entered into FUCHIA software on site (Follow-up and Care of HIV Infection and AIDS, Epicentre).

### Analysis

Categorical variables were summarised using frequency and percentage and analysed using a chi-square test. Continuous variables were first tested for normality. As all were significantly skewed they were summarised using median and inter-quartile range (IQR) and analysed using a Wilcoxon rank-sum test.

Incidence rates were designed a priori to be reported as crude, unadjusted rates. Incidence rates for any WHO stage 3 or 4 condition were calculated for five periods after ART initiation (0–3, 4–6, 7–12, 13–24 and 25–36 months) by dividing the number of occurrences of any stage 3 or 4 condition by the number of person-years at risk, within that particular time period. Patients were censored at death, loss to follow-up, or, where no disease event was recorded within a particular time period, at 3, 6, 12, 24 or 36 months respectively after ART initiation. Patients missing an appointment by ≥2 months were considered lost to follow-up and censored on the day of their last visit. All incidence estimates are conditional on patients being alive and on treatment at the beginning of the period, and as they are crude and unadjusted, and the groups are not independent, direct comparisons between time-periods should be interpreted with caution. As we did not evaluate whether incidence rates might have declined due to deaths in the prior time period, we cannot state the degree to which changing rates are due to the impact of ART or to the death of severely ill persons in the prior time period.

Disease specific incidence rates were calculated for the same time periods by dividing the number of first diagnoses of each WHO stage 3 or 4 condition by the number of person- years at risk. These analyses considered only the WHO stage 3 or 4 condition of interest and data was censored as described above for any WHO stage 3 or 4 incidence.

Analyses were also performed by region (Africa and Asia) and by age group. Data from Moldova (Eastern Europe) was excluded from the regional analysis due to the small number of patients. Confidence intervals were calculated for all rates.

Data were extracted from individual program FUCHIA databases, de-identified and analysed using Stata version 12 (StataCorp, College Station, Texas, USA).

## Results

### Patients

A cohort of 36,664 patients initiated ART treatment. Table 1 shows the baseline characteristics of 34,749 patients who contributed at least 3 months follow-up and were included in the analysis. Sixty-two percent of these patients were from African programmes and 36% were from Asian programmes. Eighty-nine percent of patients were aged ≥15 years, 5% were aged 5–14 years, and 6% were aged <5 years; there were proportionally less adults and more children in African compared to Asian programmes (p<0.001). The median time on ART was 1.33 years, with patients attending Asian programs followed longer than those attending African programmes (median 1.6 years and 1.1 years, respectively, p<0.001). A larger proportion of patients attending African sites was female (67% compared to 44%, p<0.001). The median CD4 level at ART commencement was higher in African patients than in Asian patients for all age-groups (p<0.001).

**Table 1:** Characteristics of study participants.

	Overall	Africa	Asia	p value (Africa vs. Asia)
Number of participants (%)	34,749 (100)	21, 643 (62.3)	12,506 (36.0)	
Median years of follow-up (IQR)	1.33 (0.51–2.41)	1.09 (0.41–2.38)	1.60 (0.83–2.48)	<0.001
Male Sex (%)	14,594 (42)	7,207 (33.3)	6,978 (55.8)	<0.001
Median Age (years) (IQR)	32.9 (25.7–39.2)	33.2 (25.3–40.7)	32 (26.8–37.7)	<0.001
Number of patients ≥15 years (%)	30,803 (88.6)	18,699 (86.4)	11,543 (92.3)	<0.001
Number of patients 5–14 years (%)	1,813 (5.2)	1,234 (5.7)	575 (4.6)	<0.001
Number of patients <5 years (%)	2,133 (6.2)	1,710 (7.9)	388 (3.1)	<0.001
**Median CD4 value at ART commencement (cells/mm3) (IQR)**				
Patients <5 years (n = 1999)	374 (179, 585)	568.5 (358, 981)	323 (163, 511)	<0.001
Patients 5–14 years (n = 1720)	182.5 (53.5, 366.5)	459 (177, 643)	153 (48, 272.5)	<0.001
Patients ≥15 (n = 26774)	124 (86, 172)	137 (98, 189)	103 (37–195)	<0.001

A further 600 patients with 3 months follow-up from the Moldova programme in Eastern Europe were included in all analyses except the regional analysis. Ninety-three percent of Moldovan patients were aged ≥15 years, 0.1% were aged 5–14 years and 5.8% were aged <5 years. The median time on ART was 0.58 years, 67.8% of patients were male and the median CD4 level at ART commencement for adults was 385 (IQR: 201–575) cells/mm^3^.

### Incidence of any WHO Stage 3 or 4 Conditions

During follow-up there were 19,156 diagnoses of WHO stage 3 conditions and 4,972 diagnoses of WHO Stage 4 conditions. The overall incidence of any WHO stage 3 or 4 condition was 40.02 (95% CI 39.52, 40.53) events per 100 person-years. The overall incidence for any WHO stage 3 condition was 31.77 (95% CI 31.32, 32.23) episodes per 100 person-years. This reduced by 97% from 77.81 (95% CI 75.63, 80.04) episodes per 100 person-years in the first 3 months to 2.40 (95% CI 2.24, 2.58) episodes per 100 person-years in months 25–36. The overall incidence for any WHO stage 4 condition was 8.25 (95% CI 8.02, 8.48) episodes per 100 person-years. This reduced by 98% over the 3-year follow-up, from 28.70 (95% CI 27.38, 30.07) episodes per 100 person-years in the first 3 months to 0.64 (95% CI 0.56, 0.73) episodes per 100 person-years in months 25–36.

The incidence of any WHO stage 3 condition diagnosed over follow up was similar across all age groups [Stage 3 conditions: age<5 years –34.09 episodes per 100 person-years (95% CI 31.44, 36.90); age 5–14 years –28.46 episodes per 100 person-years (95% CI 25.84, 31.27); age ≥15 years –30.59 episodes per 100 person-years (95% CI 30.14, 31.05), p = 0.24]. For WHO stage 4 conditions, the incidence in patients ≥15 years (8.21 episodes per 100 person-years; 95% CI 7.98, 8.45) and patients 5–14 years (5.73 episodes per 100 person-years; 95% CI 4.59, 6.07) was greater than that in patients <5 years (3.73 episodes per 100 person-years; 95% CI 2.89, 4.74) (p = 0.01 and 0.06 respectively). We did not detect significant differences in the incidence of any stage 4 condition between patients aged 5–14 years and patients ≥15 years (p = 0.25).

Regional differences were apparent in the overall disease burden due to both stage 3 and stage 4 conditions. The incidence of any WHO stage 3 condition was nearly 4 times higher in Africa than in Asia, with 43.98 (95% CI 43.29, 44.68) and 12.97 (95% CI 12.52, 13.42) episodes per 100 person-years respectively (p<0.001). For any WHO stage 4 condition, the overall incidence was 8.98 (95% CI 8.67, 9.30) and 7.05 (95% CI 6.73, 7.39) diagnoses per 100 person-years in Africa and Asia, respectively (p<0.001).

### Disease Specific Incidence

Tables 2 and 3 show the incidence over 3 years follow-up for new WHO stage 3 and stage 4 conditions. The maximum incidence for each stage 3 and stage 4 condition occurred during months 0–3 and decreased over time. Of the WHO stage 3 conditions, oral candidiasis and pulmonary TB had the highest initial incidence at 22.07 (95% CI 20.92, 23.28) and 21.96 (95% CI 20.81, 23.17) episodes per 100 person-years, respectively. The incidence of these conditions fell by 91% and 87%, respectively, by 3 years of follow-up. For the remaining stage 3 conditions shown, the reduction in disease specific incidence during follow-up ranged from 82% for other severe bacterial infections and weight loss >10% of body weight to 93% for unexplained anaemia, neutropenia or thrombocytopenia. Of the WHO stage 4 conditions, extra-pulmonary TB and candidiasis of the oesophagus, trachea, bronchi or lungs had the highest initial incidence, at 5.30 (95% CI 4.75, 5.90) and 4.67 (95% CI 4.16, 5.25) episodes per 100 person-years during months 0–3, respectively (Table 3). The incidence of these conditions fell by 82% and 92%, respectively, by 3 years of follow-up. For the remaining WHO stage 4 conditions shown, the reduction in disease specific incidence during follow-up ranged from 82% for mucocutaneous herpes simplex infection to 97% for nontuberculous mycobacterial infection and Pneumocystis *jiroveci* pneumonia.

**Table 2:** Disease specific incidence over 36 months for selected WHO Stage 3 conditions.

	Disease incidence following initiation of ART (no. of first diagnoses per 100 person-years; 95% CI)				
WHO stage 3 conditions	0–3 monthsn* = 34,749	4–6 monthsn* = 27,423	7–12 monthsn* = 25,868	13–24 monthsn* = 21,731	25–36 monthsn* = 12,445
Pulmonary tuberculosis	21.96 (20.81, 23.17)	9.29 (8.47, 10.18)	4.86 (4.44, 5.32)	2.74 (2.48, 3.02)	2.75 (2.41, 3.14)
Oral candidiasis	22.07 (20.92, 23.28)	8.99 (8.19, 9.88)	4.16 (3.78, 4.59)	2.20 (1.97, 2.26)	1.92 (1.64, 2.25)
Weight loss >10% of body weight	7.12 (6.49, 7.82)	2.89 (2.45, 3.41)	1.87 (1.62, 2.16)	1.32 (1.14, 1.52)	1.27 (1.05, 1.55)
Unexplained chronic diarrhoea >1 month	5.47 (4.92, 6.09)	2.83 (2.40, 3.34)	1.36 (1.15, 1.62)	0.72 (0.59, 0.87)	0.87 (0.69, 1.10)
Oral hairy leucoplakia	6.74 (6.12, 7.42)	3.51 (3.02, 4.07)	1.18 (0.98, 1.41)	0.70 (0.58, 0.85)	1.09 (0.89, 1.35)
Severe bacterial pneumonia	3.19 (2.78, 3.67)	1.65 (1.33, 2.05)	1.17 (0.97, 1.40)	0.79 (0.66, 0.95)	0.36 (0.25, 0.52)
Other severe bacterial infections (Pyomyositis)	2.12 (1.79, 2.52)	1.18 (0.91, 1.53)	0.84 (0.67, 1.04)	0.51 (0.41, 0.64)	0.38 (0.26, 0.54)
Unexplained prolonged fever >1 month	3.08 (2.67, 3.55)	1.00 (0.75, 1.32)	0.84 (0.67, 1.04)	0.46 (0.37, 0.59)	0.34 (0.23, 0.49)
Unexplained anaemia/neutropenia/thrombocytopenia	1.33 (1.07, 1.65)	0.47 (0.31, 0.70)	0.34 (0.24, 0.48)	0.18 (0.13, 0.27)	0.09 (0.04, 0.18)
Acute necrotizing ulcerativestomatitis/gingivitis/periodontitis	0.4 (0.27, 0.60)	0.24 (0.14, 0.43)	0.12 (0.07, 0.22)	0.05 (0.03, 0.11)	0.09 (0.04, 0.18)
Moderate malnutrition	0.29 (0.18, 0.46)	0.12 (0.05, 0.27)	0.07 (0.03, 0.15)	0.03 (0.01, 0.08)	0.04 (0.01, 0.12)
HIV associated chronic lung disease	n/a	0.02 (0.00, 0.14)	0.02 (0.01, 0.08)	0.01 (0.00, 0.05)	n/a
Lymphoid interstitial pneumonitis	0.03 (0.01, 0.13)	0.02 (0.00, 0.14)	0.02 (0.01, 0.08)	n/a	n/a
Lymphnode TB	0.06 (0.02, 0.17)	0.02 (0.00, 0.14)	0.03 (0.01, 0.10)	0.01 (0.00, 0.05)	0.01 (0.00, 0.09)

* n = the number of patients who were followed up for the entire time period indicated.

**Table 3:** Disease specific incidence over 36 months for selected WHO Stage 4 conditions.

	Disease incidence following initiation of ART(no. of first diagnoses per 100 person-years; 95% CI)				
WHO stage 4 conditions	0–3 monthsn* = 34,749	4–6 monthsn* = 27,423	7–12 monthsn* = 25,868	13–24 monthsn* = 21,731	25–36 monthsn* = 12,445
Candidiasis of the oesophagus,trachea, bronchi or lungs	4.67 (4.16, 5.25)	1.26 (0.98, 1.62)	0.74 (0.59, 0.94)	0.47 (0.37, 0.60)	0.38 (0.26, 0.54)
HIV wasting syndrome	2.64 (2.26, 3.08)	0.83 (0.61, 1.13)	0.47 (0.36, 0.63)	0.27 (0.20, 0.37)	0.16 (0.09, 0.28)
Extra-pulmonary tuberculosis	5.30 (4.75, 5.90)	2.63 (2.21, 3.12)	1.19 (0.99, 1.43)	0.83 (0.69, 0.99)	0.95 (0.76, 1.20)
Herpes simplex virus infectionmucocutaneous >1 month	1.44 (1.17, 1.77)	1.16 (0.89, 1.50)	0.31 (0.22, 0.44)	0.15 (0.10, 0.23)	0.26 (0.17, 0.40)
Kaposi’s sarcoma	1.07 (0.84, 1.36)	0.75 (0.55, 1.04)	0.33 (0.23, 0.47)	0.12 (0.08, 0.19)	0.13 (0.07, 0.23)
Cryptococcosis - extrapulmonary	2.82 (2.43, 3.27)	0.94 (0.70, 1.25)	0.22 (0.14, 0.33)	0.19 (0.13, 0.28)	0.18 (0.10, 0.30)
Pneumocystis *jiroveci* pneumonia	1.72 (1.42, 2.08)	0.35 (0.21, 0.56)	0.21 (0.13, 0.32)	0.16 (0.10, 0.24)	0.05 (0.02, 0.13)
Toxoplasmosis of the brain	1.47 (1.20, 1.81)	0.83 (0.61, 1.13)	0.18 (0.11, 0.28)	0.15 (0.10, 0.23)	0.06 (0.03, 0.15))
Encephalopathy	0.36 (0.23, 0.54)	0.12 (0.05, 0.27)	0.07 (0.03, 0.15)	0.05 (0.03, 0.11)	0.01 (0.00, 0.09)
Cytomegalovirus disease of anorgan other than liver, spleen orlymph nodes	0.49 (0.34, 0.69)	0.47 (0.31, 0.70)	0.25 (0.17, 0.37)	0.12 (0.08, 0.19)	n/a
Lymphoma	0.13 (0.06, 0.26)	0.06 (0.02, 0.19)	0.03 (0.01, 0.10)	0.03 (0.01, 0.07)	n/a
Cryptosporidiosis with diarrhea >1 month	0.16 (0.09, 0.30)	0.02 (0.00, 0.14)	0.02 (0.01, 0.08)	0.01 (0.00, 0.05)	0.03 (0.01, 0.10)
Non TB mycobacterial infection	3.03 (2.63, 3.50)	0.94 (0.70, 1.25)	0.27 (0.18, 0.39)	0.12 (0.08, 0.19)	0.09 (0.04, 0.18)
Any disseminated endemic mycosis	0.49 (0.34, 0.69)	0.20 (0.11, 0.38)	0.09 (0.05, 0.18)	0.02 (0.01, 0.06)	0.03 (0.01, 0.10)
Penicilliosis Marneffei	1.15 (0.91, 1.45)	0.24 (0.14, 0.43)	0.07 (0.03, 0.15)	0.01 (0.00, 0.05)	0.03 (0.01, 0.10)
Isosporidiosis with diarrhea >1 month	0.06 (0.02, 0.17)	0.12 (0.05, 0.27)	0.05 (0.02, 0.12)	0.03 (0.01, 0.08)	n/a
Septicaemia recurrent	0.03 (0.01, 0.13)	n/a	0.02 (0.01, 0.08)	n/a	n/a
Cervical carcinoma	0.06 (0.02, 0.17)	0.06 (0.02, 0.19)	0.01 (0.00, 0.07)	0.01 (0.00, 0.05)	n/a
HIV associated cardiomyopathy	0.05 (0.02, 0.15)	n/a	0.02 (0.01, 0.08)	0.02 (0.01, 0.06)	n/a
Visceral leishmaniasis	0.03 (0.01, 0.13)	0.33 (0.20, 0.53)	0.28 (0.19, 0.41)	0.12 (0.07, 0.19)	0.16 (0.09, 0.28)

* n = the number of patients who were followed up for the entire time period indicated.

### Regional Differences in Disease Specific Incidence

The disease specific incidence of individual WHO stage 3 conditions in months 0–3 was higher in Africa than in Asia for all conditions except oral hairy leucoplakia (Table 4). In Africa, pulmonary TB had the highest incidence of all WHO stage 3 conditions and this was 3 times greater than the incidence in Asia, with 30.45 (95% CI 28.68, 32.33) compared to 9.90 (95% CI 8.73, 11.23) episodes per 100 person-years in months 0–3. The incidence of oral candidiasis and weight loss >10% of body weight were also high in Africa with 29.84 (95% CI 28.09, 31.70) and 11.78 (95% CI 10.71, 12.96) episodes per 100 person- years in months 0–3, respectively. In Asia, oral candidiasis had the highest incidence of all WHO stage 3 conditions, with 10.32 (95% CI 9.12, 11.67) episodes per 100 person-years in months 0–3. The incidence of oral hairy leucoplakia was also high in Asia, with 9.41 (95% CI 8.27, 10.71) episodes per 100 person-years in months 0–3.

**Table 4:** Regional disease specific incidence of WHO stage 3 conditions in months 0–3 following ART initiation.

	Africa		Asia		
WHO stage 3 conditions	Numberof first diagnoses	Incidence in months 0–3 following initiation of ART(no. of first diagnoses per100 person-years; 95% CI)	Numberof first diagnoses	Incidence in months 0–3 following initiation of ART(no. of first diagnoses per100 person-years; 95% CI)	p value (Africa vs Asia)
Pulmonary tuberculosis	1073	30.45 (28.68, 32.33)	243	9.90 (8.73, 11.23)	<0.001
Oral candidiasis	1054	29.84 (28.09, 31.70)	253	10.32 (9.12, 11.67)	<0.001
Weight loss >10% of body weight	423	11.78 (10.71, 12.96)	13	0.52 (0.30, 0.90)	<0.001
Unexplained chronic diarrhoea >1 month	308	8.55 (7.64, 9.56)	26	1.05 (0.71, 1.54)	<0.001
Oral hairy leucoplakia	172	4.76 (4.10, 5.53)	231	9.41 (8.27, 10.71)	<0.001
Severe bacterial pneumonia	154	4.26 (3.64, 4.99)	30	1.21 (0.85, 1.73)	<0.001
Other severe bacterial infections(Pyomyositis)	113	3.12 (2.60, 3.75)	12	0.48 (0.27, 0.85)	<0.001
Unexplained prolonged fever >1 month	105	2.90 (2.40, 3.51)	67	2.70 (2.13, 3.44)	0.677
Unexplained anaemia/neutropenia/thrombocytopenia	45	1.24 (0.93, 1.66)	7	0.28 (0.13, 0.59)	<0.001
Acute necrotizing ulcerative stomatitis/gingivitis/periodontitis	23	0.63 (0.42, 0.95)	1	0.04 (0.01, 0.29)	0.003
Moderate malnutrition	17	0.47 (0.29, 0.75)	1	0.04 (0.01, 0.29)	0.002

The disease specific incidence of WHO stage 4 conditions in months 0–3 was higher in Asia for 60% of conditions (Table 5). These included extra-pulmonary TB, non tuberculous mycobacterial infections, toxoplasmosis, *pneumocystis jiroveci* pneumonia and cryptococcosis. Penicilliosis occurred exclusively in Asia. HIV wasting syndrome, candidiasis of the oesophagus, trachea, bronchi or lungs, chronic mucocutaneous herpes simplex virus infection, Kaposi’s sarcoma, encephalopathy and lymphoma were the WHO stage 4 conditions with higher incidence in Africa than Asia.

**Table 5:** Regional disease specific incidence of WHO stage 4 conditions in months 0–3 following ART initiation.

	Africa		Asia		
WHO stage 4 conditions	Numberof first diagnoses	Incidence in months 0–3 following initiation of ART(no. of first diagnoses per100 person-years; 95% CI)	Number of first diagnoses	Incidence in months 0–3 following initiation of ART(no. of first diagnoses per100 person-years; 95% CI)	p value (Africa vs Asia)
Candidiasis of the oesophagus, trachea, bronchi or lungs	208	5.76 (5.03, 6.60)	77	3.11 (2.49, 3.89)	<0.001
HIV wasting syndrome	143	3.95 (3.36, 4.66)	17	0.68 (0.43, 1.10)	<0.001
Extra-pulmonary tuberculosis	123	3.40 (2.85, 4.06)	195	7.92 (6.89, 9.12)	<0.001
Herpes simplex virus infection mucocutaneous >1 month	79	2.18 (1.75, 2.72)	9	0.36 (0.19, 0.70)	<0.001
Kaposi’s sarcoma	64	1.77 (1.38, 2.26)	2	0.08 (0.02, 0.32)	<0.001
Cryptococcosis - extrapulmonary	63	1.74 (1.36, 2.23)	107	4.33 (3.58, 5.23)	<0.001
Pneumocystis *jiroveci* pneumonia	48	1.32 (1.00, 1.76)	56	2.26 (1.74, 2.94)	0.006
Toxoplasmosis of the brain	26	0.72 (0.49, 1.05)	63	2.54 (1.99, 3.26)	<0.001
Encephalopathy	19	0.52 (0.33, 0.82)	3	0.12 (0.04, 0.37)	0.010
Cytomegalovirus disease of an organother than liver, spleen or lymph nodes	14	0.39 (0.23, 0.65)	16	0.64 (0.40, 1.05)	0.172
Lymphoma	7	0.19 (0.09, 0.40)	1	0.04 (0.01, 0.29)	0.108
Cryptosporidiosis with diarrhea >1 month	5	0.14 (0.06, 0.33)	5	0.20 (0.08, 0.48)	0.571
Non TB mycobacterial infection	3	0.08 (0.03, 0.26)	184	7.47 (6.47, 8.64)	<0.001
Any disseminated endemic mycosis	2	0.06 (0.01, 0.22)	28	1.13 (0.78, 1.64)	<0.001
Penicilliosis Marneffei	0	n/a	71	2.87 (2.27, 3.62)	n/a

These regional differences in disease specific incidence continued over time, but by 24–36 months follow-up post ART initiation, the only differences between regions were for the WHO stage 3 conditions pulmonary TB, oral candidiasis, weight loss and chronic diarrhoea which remained higher in Africa than Asia.

### Age Differences in Disease Specific Incidence

In adults and children aged 5–14 years, pulmonary TB had the highest incidence of all WHO stage 3 or 4 conditions in months 0–3, with 22.24 (95% CI 21.02, 23.53)and 25.76 (95% CI 20.67, 32.12) episodes per 100 person-years, respectively (Table 6 and Table 7). The incidence of oral candidiasis, oral leucoplakia and weight loss greater than 10% of body weight were also high in adults. In children aged 5–14 years the incidence of oral candidiasis was highest in months 0–3 with severe bacterial pneumonia becoming the most common in months 13–24 [incidence 5.07 (95% CI 3.34, 7.69)]. For children <5 years, oral candidiasis had the highest incidence in months 0–3 (25.79 episodes per 100 person-years), with pulmonary TB taking precedence after 12 months ([incidence 4.76 (95% CI 2.38, 9.51) in months 13–24]. Other conditions with high incidence in children <5 years were weight loss greater than 10% of body weight and unexplained chronic diarrhoea for more than month.

**Table 6:** Disease specific Incidence of WHO stage 3 conditions in months 0–3 following ART initiation by age.

WHO stage 3 conditions	Age <5 years		Age 5–14 years		Age ≥15 years	
	Numberof first diagnoses	Incidence in months0–3 following initiationof ART(no. of first diagnoses per 100 person-years; 95% CI)	Numberof first diagnoses	Incidence in months0–3 following initiationof ART(no. of first diagnoses per 100 person-years; 95% CI)	Numberof first diagnoses	Incidence in months0–3 following initiationof ART(no. of first diagnoses per 100 person-years; 95% CI)
Pulmonary tuberculosis	51	14.33 (10.89, 18.85)	79	25.76 (20.67, 32.12)	1199	22.24 (21.02, 23.53)
Oral candidiasis	91	25.79 (21.00, 31.67)	49	15.82 (11.95, 20.91)	1197	22.19 (20.96, 23.48)
Oral hairy leucoplakia	5	1.39 (0.58, 3.33)	8	2.55 (1.28, 5.11)	401	7.33 (6.64, 8.08)
Weight loss >10% of body weight	36	10.06 (7.26, 13.95)	14	4.48 (2.65, 7.56)	388	7.08 (6.41, 7.82)
Unexplained chronic diarrhoea >1 month	22	6.12 (4.03, 9.29)	19	6.08 (3.88, 9.53)	296	5.40 (4.81, 6.05)
Unexplained prolonged fever >1 month	5	1.39 (0.58, 3.33)	11	3.52 (1.95, 6.35)	174	3.17 (2.73, 3.67)
Severe bacterial pneumonia	19	5.29 (3.37, 8.29)	24	7.71 (5.16, 11.50)	154	2.80 (2.39, 3.28)
Other severe bacterial infections (Pyomyositis)	6	1.67 (0.75, 3.71)	9	2.88 (1.50, 5.53)	116	2.11 (1.76, 2.53)
Unexplained anaemia/neutropenia/thrombocytopenia	3	0.83 (0.27, 2.58)	6	1.92 (0.86, 4.26)	73	1.33 (1.05, 1.67)
Acute necrotizing ulcerativestomatitis/gingivitis/periodontitis	2	0.55 (0.14, 2.21)	2	0.64 (0.16, 2.55)	21	0.38 (0.25, 0.58)
HIV associated chronic lung disease	0	n/a	0	n/a	0	n/a
Lymphoid interstitial pneumonitis	0	n/a	2	0.64 (0.16, 2.55)	0	n/a
Lymphnode TB	1	0.28 (0.04, 1.97)	3	0.96 (0.31, 2.97)	n/a	n/a
Moderate malnutrition	13	3.62 (2.10, 6.23)	5	1.60 (0.66, 3.83)	n/a	n/a

**Table 7:** Disease specific Incidence of WHO stage 4 conditions in months 0–3 following ART initiation by age.

WHO stage 4 conditions	Age <5 years		Age 5–14 years		Age ≥15 years	
	Numberof first diagnoses	Incidence in months0–3 after initiation of ART(no. of first diagnoses per 100 person-years; 95% CI)	Numberof first diagnoses	Incidence in months0–3 after initiationof ART(no. of first diagnoses per 100 person-years; 95% CI)	Numberof first diagnoses	Incidence in months 0–3 after initiation of ART(no. of first diagnoses per 100 person-years; 95% CI)
Extra-pulmonary tuberculosis	2	0.55 (0.14, 2.21)	6	1.92 (0.86, 4.26)	318	5.80 (5.20, 6.48)
Candidiasis of the oesophagus,trachea, bronchi or lungs	4	1.11 (0.42, 2.96)	7	2.23 (1.06, 4.68)	277	5.05 (4.49, 5.68)
Non TB mycobacterial infection	3	0.83 (0.27, 2.58)	4	1.28 (0.48, 3.40)	180	3.28 (2.83, 3.79)
Cryptococcosis	1	0.28 (0.04, 1.97)	0	n/a	173	3.15 (2.71, 3.65)
HIV wasting syndrome	19	5.29 (3.37, 8.29)	8	2.55 (1.28, 5.11)	136	2.47 (2.09, 2.92)
Pneumocystis *jiroveci* pneumonia	7	1.94 (0.93, 4.08)	6	1.92 (0.86, 4.26)	93	1.69 (1.38, 2.07)
Toxoplasmosis of the brain	0	n/a	1	0.32 (0.04, 2.26)	90	1.64 (1.33, 2.01)
Herpes simplex virus infectionmucocutaneous >1 month	0	n/a	4	1.28 (0.48, 3.40)	85	1.54 (1.25, 1.91)
Penicilliosis Marneffei	0	n/a	3	0.96 (0.31, 2.97)	68	1.24 (0.97, 1.57)
Kaposi’s sarcoma	0	n/a	1	0.32 (0.04, 2.26)	65	1.18 (0.93, 1.50)
Cytomegalovirus disease of an organ otherthan liver, spleen or lymph nodes	2	0.55 (0.14, 2.21)	1	0.32 (0.04, 2.26)	27	0.49 (0.34, 0.71)
Any disseminated endemic mycosis	2	0.55 (0.14, 2.21)	2	0.64 (0.16, 2.55)	26	0.47 (0.32, 0.69)
Encephalopathy	0	n/a	2	0.64 (0.16, 2.55)	20	0.36 (0.23, 0.56)
Cryptosporidiosis with diarrhea >1 month	0	n/a	1	0.32 (0.04, 2.26)	9	0.16 (0.08, 0.31)
Lymphoma	1	0.28 (0.04, 1.97)	0	n/a	7	0.13 (0.06, 0.27)
Isosporidiosis with diarrhea >1 month	0	n/a	0	n/a	4	0.07 (0.03, 0.19)
Cervical carcinoma	0	n/a	0	n/a	4	0.07 (0.03, 0.19)
Septicaemia recurrent	0	n/a	0	n/a	2	0.04 (0.01, 0.15)
Visceral leishmaniasis	0	n/a	0	n/a	2	0.04 (0.01, 0.15)
HIV associated cardiomyopathy	0	n/a	1	0.32 (0.04, 2.26)	2	0.04 (0.01, 0.15)
HIV associated nephropathy	0	n/a	0	n/a	0	n/a
Progressive multifocal leucoencephalopathy	0	n/a	0	n/a	0	n/a

Between months 0–3 and months 6–12 on ART the incidence of pulmonary TB decreased in adults by 78.5% from 22.24 (95% CI 21.02, 23.53) to 4.78 episodes per 100 person-years (95% CI 4.35, 5.25), and in children aged 5–14 years there was a 77% reduction from 25.76 (95% CI 20.67, 32.12) to 5.86 episodes per 100 person-years (95% CI 3.59, 9.56). In comparison, for children <5 years the incidence of pulmonary TB decreased by 48% from 14.33 (95% CI 10.89, 18.85) to 7.45 episodes per 100 person-years (95% CI 4.23, 13.12). In the period 6–12 months on ART, bacterial pneumonia was more commonly diagnosed in children [incidence 6.82 episodes per 100 person-years (95% CI 3.78, 12.33) for those <5 years and 5.50 episodes per 100 person-years (95% CI 3.31, 9.12) for those 5–14 years] compared to adults (incidence 0.94 episodes per 100 person-years (95% CI 0.76, 1.16).

## Discussion

We demonstrated a high overall incidence of any WHO stage 3 or 4 condition (40.02 episodes per 100 person-years) in the 3 years following ART initiation in patients in RLS. In the 3 months immediately following ART initiation, the incidence of any WHO stage 3 or 4 condition was nearly 14 times higher than that reported for a resource-rich setting by the Swiss HIV Cohort Study (106.5 episodes compared with 7.7 episodes per 100 person-years) [6]. However, we also showed that there were significant reductions, particularly in the first 12 months, in the incidence of new WHO stage 3 or 4 conditions during the first 36 months of ART. Both stage 3 and 4 conditions reduced in overall incidence by more than 90% in the first year and reductions in disease specific incidence rates of the conditions with highest initial incidence ranged from 82% to 97%. These striking decreases in incidence during the first year of ART are similar to those observed in patients on ART in high income countries [6], [9], [13]. As our analyses are not adjusted for death and loss to follow-up in the cohort, the absolute reductions in incidence rates cannot be ascribed to the beneficial effects of ART alone [14]. Nevertheless it is known that the immune restorative effects of ART reduce the incidence of new or recurrent WHO stage 3 and 4 conditions [6], [7], [8], [9] and it is likely that ART has played a significant role in their reductions in our cohort. This emphasizes the importance of access to early ART initiation for those eligible to reduce the impact of HIV related conditions during early ART in RLS. This requires increased efforts to enable early diagnosis and accurate clinical and immunological staging of HIV infected patients, and their rapid access to effective, non-toxic, free and sustained ART in these settings.

The considerable incidence of HIV associated clinical conditions during early ART also emphasizes the importance of increased disease prevention efforts prior to ART commencement. Such interventions include isoniazid chemophrophylaxis for TB [15], [16], fluconazole prophylaxis for those who are cryptococcal antigen negative and have delays in ART initiation [17], [18] and cotrimoxazole therapy for PCP, toxoplasmosis, bacterial sepsis and malaria [19], [20]. Resources and improved tools are also required to facilitate early and improved diagnosis of opportunistic infections, as well as effective, adapted and affordable treatments to minimize their impact on morbidity and mortality.

We defined the burden of different WHO stage 3 and 4 conditions stratified by age and region. This provides information for clinicians to use in determining which conditions to focus on for prevention, early diagnosis and treatment. It is also vital information for programme planners to guide allocation of resources required for disease management in patients on ART, and for strategic planning and resource prioritization. For example, age-specific and regional incidence rates of TB in patients on ART can aid strategic decision making about which patient populations to prioritise in implementing isoniazid preventive therapy (IPT) if universal IPT is not chosen or possible. Also, knowing specific disease incidences can aid in decision making about programme drug formularies.

TB and candidiasis were the most common conditions seen in our RLS cohort. TB is a leading cause of disease in HIV infected patients and is the leading cause of HIV-related deaths [2], [17], [21]. Our results re-affirm the importance of TB as a pathogen in HIV infected patients treated with ART. Importantly, our results support other studies in suggesting that ART alone reduces the incidence of TB [22], [23], [24]. Provision of IPT can further reduce the impact of TB whilst on ART [15], [25] and is currently recommended [16]. Likewise our study showed ART is associated with a reduction in the incidence of candidiasis. Although a recent study from Uganda showed that giving fluconazole prophylaxis to those with a CD4 count <200 cells/mm^3^ can further reduce the impact of candidiasis both pre and post ART [17], it did not reduce overall mortality, and is currently not recommended by WHO [18]. Important contributions to the disease burden in our study were also made by potentially preventable stage 4 conditions such as non-tuberculous mycobacteria, cryptococcosis, pneumocystis and toxoplasmosis. The most common conditions differed from those in a resource rich setting where the oseophageal candidiasis, PCP and cytomegalovirus disease were the most frequently reported conditions during the first 6 months of ART [6].

There were important regional differences in the overall and disease specific incidences of any stage 3 and 4 condition following ART initiation. The incidence of any stage 3 HIV associated condition was almost 4 times higher in Africa than Asia despite African patients being less immunosuppressed overall at baseline. This may be due to increased exposure to the relevant pathogens in Africa or less likely, to improved diagnosis in Africa [22], [26]. Some individual stage 4 conditions were more common in Asian programmes, particularly in the first 3 months after ART initiation, likely due to the higher rate of immunosuppression of these patients at baseline, and differential prevalence of specific pathogens. For example, penicilliosis is not endemic in Africa.

The incidence of any first stage 3 condition did not differ by age however there were important differences in the incidence of individual conditions by age. Pulmonary TB was diagnosed less commonly in children <5 years, although this may be underestimated given the difficulty of diagnosis in this age-group. Additionally, severe bacterial pneumonia was more common in children <15 years compared with adults.

There were significant age-related differences in the overall incidences of stage 4 conditions that may be explained by the relative level of baseline immunodeficiency. HIV-infected children <5 years usually have rapidly progressive disease with high mortality [27]. This increased immunodeficiency may explain the higher incidence of stage 4 conditions in this age-group. Conversely, many children in the 5–14 age-group will have been infected peripartum, but survived due to less rapidly progressive disease. This may explain this group’s low incidence of stage 4 conditions. In addition, there were more differences seen in the incidence of specific stage 4 conditions between children <5 years and adults. Children <5 years were more likely to have HIV wasting compared with adults but less likely to have all other conditions except pneumocystis pneumonia. There were no cases of toxoplasmosis, penicilliosis or Kaposi’s sarcoma in patients <5 years of age. As reported previously [28], [29], crytptococcosis was rare under 15 years of age (1 case), supporting recent WHO guidelines recommending against screening and pre-emptive treatment in children due to low prevalence [18].

Information about age-specific incidence rates of various conditions during ART may have important practical applications. For example, TB incidence reduced by 48% during the first 12 months of ART in children <5 years which was less than the 79% reduction in adults and 77% reduction in children 5–14 years. This suggests IPT may have relatively greater additional impact in children <5 years than older age-groups during early ART. In addition, the incidence of bacterial pneumonia during the first 12 months of ART was up to 7 times higher in children than adults, supporting particular attention being paid to the prevention, diagnosis and treatment of this condition in children.

This study has a number of limitations. Firstly, reliance on basic investigations to support clinical assessment in the diagnosis of HIV associated conditions may have resulted in diagnostic inaccuracies. For example TB in children <5 years old is difficult to confirm with AFB stains alone, and reliance on clinical assessment may have under or overestimated its true incidence. In addition, clinician practices and availability of investigations may have varied between programmes, influencing the relative frequencies of diagnoses. Nevertheless, the results of this study remain relevant because the diagnoses reflect the reality of clinical practice in most RLS where ART is delivered and involves a large cohort of patients. Secondly, the lack of pre-ART data precluded the comparison of incidence rates before and after ART. Thirdly, the Asian data was predominantly from Myanmar programmes so results may not be generalisable across all Asian programmes. Fourthly, we cannot judge the extent to which declining incidence rates are due to ART and care effects; some of these declines may be due to deaths of severely ill persons in the prior time periods. Finally, we were unable to ascertain whether patients lost to follow-up developed any HIV related conditions. Furthermore, as inclusion criteria required at least 3 months of follow-up on ART, patients developing stage 3 and 4 conditions in the first 3 months, which may have increased their risk of lost-to follow-up, could have led to an underestimation of incidence rates in this initial period.

### Conclusion

In RLS the incidence of WHO stage 3 and 4 conditions in the early period following ART initiation is high when compared to similar studies in resource-rich settings. However, with early deaths of the sickest patients and with the benefits of ART for the remaining patients, the incidence of HIV associated conditions greatly reduces with time on ART, particularly within the first 12 months. TB and candidiasis account for the greatest disease burden, but many other potentially preventable diseases are also important. Efforts directed towards prevention, early diagnosis and effective treatment of these conditions before and during ART, and the early and accurate assessment for, and initiation of, ART in eligible patients, are important to reduce morbidity during ART in RLS.

## References

[1] World Health Organisation (2010) Antiretroviral therapy for HIV infection in adults and adolescents. Recommendations for a public health approach: 2010 revision. Geneva. 23741771

[2] Lawn SD, Harries AD, Wood R (2010) Strategies to reduce early morbidity and mortality in adults receiving antiretroviral therapy in resource-limited settings. Curr Opin HIV AIDS 5: 18–26. 20046144 10.1097/COH.0b013e328333850f PMC3772276

[3] Zachariah R, Fitzgerald M, Massaquoi M, Pasulani O, Arnould L, et al (2006) Risk factors for high early mortality in patients on antiretroviral treatment in a rural district of Malawi. AIDS 20: 2355–2360. 17117022 10.1097/QAD.0b013e32801086b0

[4] Etard JF, Ndiaye I, Thierry-Mieg M, Gueye NF, Gueye PM, et al (2006) Mortality and causes of death in adults receiving highly active antiretroviral therapy in Senegal: a 7-year cohort study. AIDS 20: 1181–1189. 16691070 10.1097/01.aids.0000226959.87471.01

[5] Castelnuovo B, Manabe YC, Kiragga A, Kamya M, Easterbrook P, et al (2009) Cause-Specific Mortality and the Contribution of Immune Reconstitution Inflammatory Syndrome in the First 3 Years after Antiretroviral Therapy Initiation in an Urban African Cohort. Clinical Infectious Diseases 49: 965–972. 19673615 10.1086/605500

[6] Ledergerber B, Egger M, Erard V, Weber R, Hirschel B, et al (1999) AIDS-related opportunistic illnesses occurring after initiation of potent antiretroviral therapy: the Swiss HIV Cohort Study. JAMA 282: 2220–2226. 10605973 10.1001/jama.282.23.2220

[7] Ives NJ, Gazzard BG, Easterbrook PJ (2001) The changing pattern of AIDS-defining illnesses with the introduction of highly active antiretroviral therapy (HAART)in a London clinic. J Infect 42: 134–139. 11531320 10.1053/jinf.2001.0810

[8] Kaplan JE, Hanson D, Dworkin MS, Frederick T, Bertolli J, et al (2000) Epidemiology of human immunodeficiency virus-associated opportunistic infections in the United States in the era of highly active antiretroviral therapy. Clin Infect Dis 30 Suppl 1S5–14. 10770911 10.1086/313843

[9] Buchacz K, Baker RK, Palella FJ Jr, Chmiel JS, Lichtenstein KA, et al (2010) AIDS-defining opportunistic illnesses in US patients, 1994–2007: a cohort study. AIDS 24: 1549–1559. 20502317 10.1097/QAD.0b013e32833a3967

[10] Lynen L (2006) Clinical HIV/AIDS Care Guidelines for Resource-poor Settings, Second Edition. Médecins Sans Frontières Operational Centre Brussels. April.

[11] World Health Organisation (2006) Antiretroviral therapy for adults and adolescents in resource-limited settings:towards universal access. Geneva: World Health Organisation.

[12] World Health Organization (2004) Scaling up antiretroviral therapy in resource-limited settings: treatment guidelines for a public health approach. Geneva.

[13] d’Arminio Monforte A, Sabin CA, Phillips A, Sterne J, May M, et al (2005) The changing incidence of AIDS events in patients receiving highly active antiretroviral therapy. Archives of internal medicine 165: 416–423. 15738371 10.1001/archinte.165.4.416

[14] Hoover DR, Munoz A, Carey V, Odaka N, Taylor JM, et al (1991) The unseen sample in cohort studies: estimation of its size and effect. Multicenter AIDS Cohort Study. Statistics in medicine. 10(12): 1993–2003. 10.1002/sim.4780101212 1805323

[15] Golub JE, Pronyk P, Mohapi L, Thsabangu N, Moshabela M, et al (2009) Isoniazid preventive therapy, HAART and tuberculosis risk in HIV-infected adults in South Africa: a prospective cohort. AIDS 23: 631–636. 19525621 10.1097/QAD.0b013e328327964f PMC3063949

[16] World Health Organisation (2011) Guidelines for intensified tuberculosis case-finding and isoniazid preventive therapy for people living with HIV in resource-constrained settings. Geneva

[17] Kasprowicz VO, Achkar JM, Wilson D (2011) The tuberculosis and HIV epidemic in South Africa and the KwaZulu-Natal Research Institute for Tuberculosis and HIV. J Infect Dis 204 Suppl 4S1099–1101. 21996691 10.1093/infdis/jir414

[18] World Health Organisation (2011) Rapid advice: diagnosis, prevention and management of cryptococcal disease in HIV-infected adults, adolescents and children. 26110194

[19] Walker AS, Ford D, Gilks CF, Munderi P, Ssali F, et al (2010) Daily co-trimoxazole prophylaxis in severely immunosuppressed HIV-infected adults in Africa started on combination antiretroviral therapy: an observational analysis of the DART cohort. Lancet 375: 1278–1286. 20347483 10.1016/S0140-6736(10)60057-8 PMC2858802

[20] Mermin J, Lule J, Ekwaru JP, Malamba S, Downing R, et al (2004) Effect of co-trimoxazole prophylaxis on morbidity, mortality, CD4-cell count, and viral load in HIV infection in rural Uganda. Lancet 364: 1428–1434. 15488218 10.1016/S0140-6736(04)17225-5

[21] Sloan DJ, Dedicoat MJ, Lalloo DG (2009) Treatment of cryptococcal meningitis in resource limited settings. Curr Opin Infect Dis 22: 455–463. 19587589 10.1097/QCO.0b013e32832fa214 PMC3977571

[22] World Health Organisation (2011) Global tuberculosis control. World Health Organisation.

[23] Miranda A, Morgan M, Jamal L, Laserson K, Barreira D, et al (2007) Impact of antiretroviral therapy on the incidence of tuberculosis: the Brazilian experience, 1995–2001. PLoS One 2: e826. 17786198 10.1371/journal.pone.0000826 PMC1952142

[24] Manosuthi W, Chottanapand S, Thongyen S, Chaovavanich A, Sungkanuparph S (2006) Survival rate and risk factors of mortality among HIV/tuberculosis-coinfected patients with and without antiretroviral therapy. Journal of acquired immune deficiency syndromes 43: 42–46. 16885778 10.1097/01.qai.0000230521.86964.86

[25] Fielding K, Grant A, Lewis J, Hayes R, Churchyard G (2012) Individual-level effect of isoniazid preventive therapy on risk of TB: the Thibela TB Study. 19th Conference on Retroviruses and Opportunistic Infections Seattle, USA.

[26] UNICEF/WHO (2006) Pneumonia: The forgotten killer of children.

[27] Newell M-L, Coovadia H, Cortina-Borja M, Rollins N, Gaillard P, et al (2004) Mortality of infected and uninfected infants born to HIV-infected mothers in Africa: a pooled analysis. The Lancet 364: 1236–1243. 10.1016/S0140-6736(04)17140-7 15464184

[28] Gonzalez CE, Shetty D, Lewis LL, Mueller BU, Pizzo PA, et al (1996) Cryptococcosis in human immunodeficiency virus-infected children. The Pediatric infectious disease journal 15: 796–800. 8878224 10.1097/00006454-199609000-00012

[29] Abadi J, Nachman S, Kressel AB, Pirofski L (1999) Cryptococcosis in children with AIDS. Clinical infectious diseases : an official publication of the Infectious Diseases Society of America 28: 309–313. 10064249 10.1086/515130

